# The effect of feature-based attention on flanker interference processing: An fMRI-constrained source analysis

**DOI:** 10.1038/s41598-018-20049-1

**Published:** 2018-01-25

**Authors:** Julia Siemann, Manfred Herrmann, Daniela Galashan

**Affiliations:** 1 Department of Neuropsychology and Behavioral Neurobiology, Center for Cognitive Sciences (ZKW), University of Bremen, Bremen, Germany; 2 Center for Advanced Imaging (CAI), University of Bremen, Bremen, Germany; 3 Department of Medical Psychology and Medical Sociology, University Hospital Schleswig-Holstein, Kiel, Germany

## Abstract

The present study examined whether feature-based cueing affects early or late stages of flanker conflict processing using EEG and fMRI. Feature cues either directed participants’ attention to the upcoming colour of the target or were neutral. Validity-specific modulations during interference processing were investigated using the N200 event-related potential (ERP) component and BOLD signal differences. Additionally, both data sets were integrated using an fMRI-constrained source analysis. Finally, the results were compared with a previous study in which spatial instead of feature-based cueing was applied to an otherwise identical flanker task. Feature-based and spatial attention recruited a common fronto-parietal network during conflict processing. Irrespective of attention type (feature-based; spatial), this network responded to focussed attention (valid cueing) as well as context updating (invalid cueing), hinting at domain-general mechanisms. However, spatially and non-spatially directed attention also demonstrated domain-specific activation patterns for conflict processing that were observable in distinct EEG and fMRI data patterns as well as in the respective source analyses. Conflict-specific activity in visual brain regions was comparable between both attention types. We assume that the distinction between spatially and non-spatially directed attention types primarily applies to temporal differences (domain-specific dynamics) between signals originating in the same brain regions (domain-general localization).

## Introduction

The term ‘attention’ describes the concentration of available resources on a subset of all incoming information units at a given time. Both spatial and non-spatial information (such as object features) can serve as entities to guide attention.^1^

On a neuronal level, spatial attention is characterised by enhanced responses of neurons preferentially processing attended locations^2^. Similarly, attention to a certain feature increases neuronal responses in brain regions processing that feature irrespective of the stimulus location. Thus, neurons in extrastriate cortical regions respond more strongly to a spatially ignored stimulus if its colour matches the currently attended colour^3^. Increased neuronal activity due to feature-based attention therefore depends on the correspondence between a neuron’s preferred feature and the currently attended feature^4^. Accordingly, selection based on features is assumed to affect the entire visual field by initiating both neuronal enhancement of attended features and suppression of simultaneously presented ignored features^5,6^. In addition to such response gain changes, a competitive interaction of stimuli simultaneously presented within a neuron’s receptive field is suggested, where attention to one stimulus enhances its response gain and simultaneously suppresses the gain of the unattended stimulus^7^. In this so-called ‘biased competition model’^8^ it is assumed that neuronal signals evoked by external stimuli are compared with a top-down attentional template that represents the current task demands. In this model attention hence causes a top-down bias of the visual focus towards the target stimulus.

In the present study we examined with fMRI and EEG how top-down feature-based cueing influences interference processing. Both methods were also integrated in an fMRI-constrained source analysis approach. Furthermore, the current data are compared afterwards to results of a previous study using spatial instead of feature-based cueing with an otherwise analogous flanker design^9^.

In the literature on feature-based attention, two competing theories have been established that lead to distinct predictions concerning the present research question. According to the ‘feature-integration theory’ simple features are initially processed in parallel to create feature maps separately for each feature dimension (e.g. colour; orientation), whereas subsequent binding of features to objects at specific locations requires serial scanning of the single feature conjunctions^10^. This theory suggests that simple feature detection can be achieved in parallel, independent of the number of items, whereas searching for feature conjunctions involves serial scanning of single item until the target is found^10^. The feature integration theory represents a hierarchical model with a superior role for spatial selection. This idea would imply that feature-based cueing of the correct stimulus colour does not affect early interference processing to the same extent as in our previous study using spatial cueing because all features (attended and unattended) are initially processed in parallel.

However, according to the alternative ‘guided search model’ early parallel processing involves the creation of feature maps that include both location information and the behavioural saliency of each feature^11^. Thus, behaviourally relevant features may guide the transfer of information from the first to the second stage of information processing to facilitate the selection of relevant information. In contrast to the feature integration model, guided search allows early attentional engagement^11^. Following this alternative model, reduced interference effects should be expected even when using non-spatial colour cues.

Summing up, with this study we can determine whether the feature-integration theory or the guided search model deliver the best explanation of the present data.

Usually, attentional mechanisms elicit activity in fronto-parietal brain areas. In more detail, for attentional focusing activities are mainly found in the dorsal fronto-parietal network^12,13^ whereas reorienting rather activates the ventral fronto-parietal network^13,14^. In EEG studies, attention effects are expressed in higher N2b amplitudes for attended stimuli (e.g.^15,16^) and in N2 modulations for feature-based attention^16^. Feature-based attention also evokes the so called selection negativity (SN) component within the time range of the N2^15^.

Complementary to attentional top-down influences as described before, interference tasks like the Eriksen flanker task^17^ demonstrate that bottom-up factors such as stimulus saliency also lead to biases initiating an involuntary attentional shift^18^. As the flanker effect indicates the selectivity of target processing^19^, it is closely linked to attentional selection and the suppression of irrelevant information. Brain areas usually involved in flanker interference are prefrontal regions and anterior cingulate cortex (ACC)^20–22^. Indeed, a meta-analysis found common activity for flanker tasks in right insula and right dorsolateral prefrontal cortex and not in ACC^23^. A network comprising parietal, frontal and cingulate areas seems to be involved in executive control not only in the flanker task^14,24^. Within this network frontal areas might be involved mainly when top-down control is needed whereas parietal parts might be concerned with stimulus saliency processing^25^.

With respect to the EEG data, an enhanced fronto-central N2 is reported for incompatible compared to compatible flanker stimuli^26,27^. This component is assumed to have its origin in anterior cingulate cortex (ACC^28,29^). In sum, feature-based attention and flanker congruency both lead to negative ERP deflections around 250 ms (selection negativity and N200 respectively^30,31^).

Concerning the interaction of selective attention and interference, several studies show that focused spatial attention can reduce the extent to which interfering information is processed^32–35^. For example, in our previous study, we investigated whether correctly directed spatial attention (valid cueing) can successfully alter the weighting of these two sources of bias (attentional cueing, interference). For this purpose, validly cued flanker trials were compared with invalidly and neutrally cued trials. We found strong evidence for early modulations of neuronal flanker processing with spatial attention, suggesting an advantage based on attentional focusing^9^.

In the present study, we used identical flanker stimuli, timing, and stimulus sequences as in our previous spatial flanker study but with colour cues. Thereby we could examine if globally directed feature-based attention reduces interference processing in a comparable way as spatial attention does. To our knowledge, the effects of feature-based attention on interference processing have not been addressed in past research. We have to note that the flanker paradigm itself includes a spatial aspect as the distracting flanker stimuli are arranged laterally next to the target stimulus and participants knew they had to respond to the central target letter and ignore the flankers. Therefore, spatial selection should also be involved to solve the present task. Nevertheless, the type of attentional orienting applied was feature-based and the behavioural data were expected to show benefits for valid as well as costs for invalid feature cues.

Furthermore, the question whether spatial and non-spatial attentional selection mechanisms are arranged hierarchically or work in concert to achieve optimal information processing has been raised repeatedly in the literature^2,36,37^. Therefore, a comparison of the current results with feature-based cueing and the results of our previous study with spatial cueing^9^ using an otherwise identical task can shed light on comparable and distinct processing mechanisms across domains. When feature-based and spatial attention are compared with fMRI, common activations are found in frontal^38^ as well as in parietal areas^36,39,40^.

Indeed, some authors report temporal differences between spatial and feature-based attentional processing. For example, there is behavioural^41^ as well as electrophysiological evidence for earlier deployment of spatial attention^42^. In contrast, Hopf and colleagues^43^ observed an initial feature-based selection ~30 ms before the spatial focusing onto the target stimulus. In the latter study the source of the first (selection) process also showed another distribution with a more posterior focus even though both processes originated in ventral occipito-temporal brain areas.

Differing stimulus probabilities influence several effects we examined in the current study, e.g. the size of the flanker effect (see ref.^44^), the N200 component (see refs^45,46^), and activity in fronto-parietal areas (see refs^13,47^). Therefore, all experimental conditions were presented with equal probabilities in order to separate effects caused by attentional cueing and interference from effects solely induced by differing stimulus frequencies.

## Hypotheses of the present study

The present study examined the effect of feature-based cueing on flanker conflict with EEG and fMRI. Data from both methods were analysed in an fMRI-constrained source analysis approach. Feature cues predicted the colour of flanker stimuli either correctly (valid cueing), incorrectly (invalid cueing), or contained no predictive value (neutral cueing). All three validities had equal probabilities (each type of cue was presented on 1/3 of all trials).

With respect to reaction times and error rates we expected main effects of feature-based attention (invalid > neutral > valid) and flanker interference (incongruent > congruent), as well as an interaction of both factors with reduced interference for valid cueing. An alternative hypothesis would expect additive effects on a behavioural level^48^ due to temporally differing processing of spatial and feature-based attention^42,43^.

As feature-based and spatial attention share common fronto-parietal brain regions, for the fMRI data we expected fronto-parietal activity patterns analogous to those reported in Siemann *et al*.^9^. A derived hypothesis is therefore an interaction of both factors (feature-based attention and interference processing).

Concerning EEG data we hypothesised effects in the N200 time window yielding reduced interference under valid cueing.

The feature-integration theory does not predict an influence of feature-based cueing on interference processing as pronounced as in our previous spatial study. The guided search model instead predicts a modulation caused by feature cues. Hence, we will examine if the data are in better agreement with the one or the other theoretical account.

## Material and Methods

### Study participants

Data from 21 healthy, right-handed (median: 100%; range = 77–100% according to the Edinburgh Inventory^49^) volunteers were obtained (10 male; mean age = 24.9 years; standard deviation (SD) = 3). All participants had normal or corrected-to-normal vision and did not show any signs of colour-blindness (tested with a modified version of the Ishihara Test^50^ including the colours from the current experiment). Each volunteer absolved a training session and took part in an EEG experiment and an MRI experiment (on separate days).

The study protocol was approved by the ethics committee of the University of Bremen and held in line with the Helsinki Declaration of the World Medical Association^51^. Written and informed consent was obtained from all volunteers before their participation in the experiments. Participants were told that they could quit the experiment anytime without giving reasons. The volunteers were given course credits or received 30€ financial compensation.

### Experimental procedure

**Figure 1:**
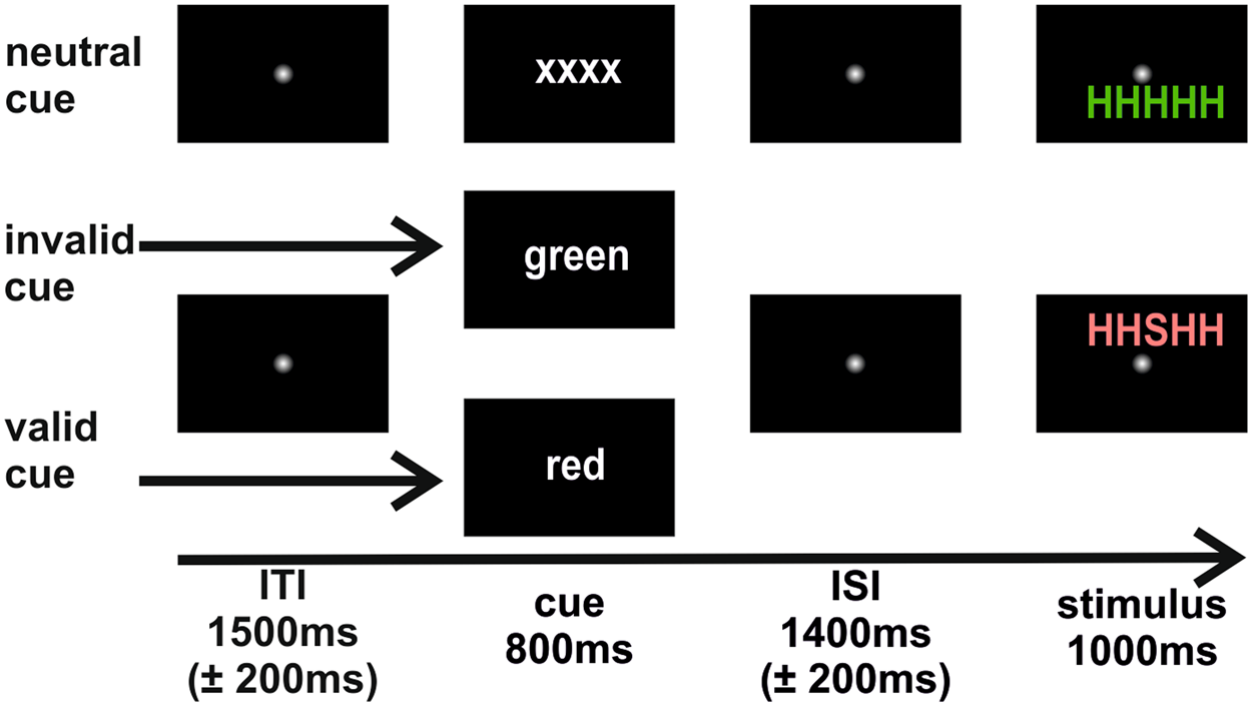
Types of trials with timing from EEG sessions. Following a jittered intertrial interval, upcoming stimulus colour was cued (red, green, uninformative). After the interstimulus interval Flanker stimuli were presented either in green or red above or below fixation. Participants responded to the identity of the central letter. Top: Neutral cueing; Middle: Invalid cueing; Bottom: Valid cueing.

Apart from the type of cueing the experimental procedure was analogous to a previous study^9^. Congruent (CON) and incongruent (INC) stimuli (target letter ‘H’/‘S’ flanked by four identical/opposite letters respectively) were presented in red or green hue above or below a fixation point (see Fig. 1). Cues either predicted a stimulus appearing in red colour (‘rot’; 1.3 × 1.2°) or green colour (‘grün’; 3.1 × 1.25°), or no relevant hue information on neutral trials (‘xxxx’; 3.2 × 0.9°). This design added up to six experimental conditions that appeared with equal probabilities in five runs. Hence, in 1/3 of the trials the cue was valid, in another 1/3 of trials invalid and the remaining 1/3 of trials had a neutral cue (overall cue frequency = 33.33%, validity of informative cues = 50%). Congruency had a probability of 50% as half of the trials showed congruent flankers and the other half incongruent flankers (equally distributed over trials with valid, invalid and neutral cues).

Participants were instructed to use the cueing information and to expect the stimulus array in the cued colour. They were asked to respond to the identity of the central letter using their right index and middle fingers. Stimulus-finger-mapping was counterbalanced across all participants.

Each volunteer completed three separate sessions. During the training session participants were familiarised with the experimental sequence using a standardised written task instruction. Participants were informed about the equiprobable cue validities. In Fig. 1 sample trials are shown for the three cue validity levels.

EEG and fMRI sessions were measured on separate days (mean time span between both measurements: 2.7 days; SD = 2.4). Half of the female as well as half of the male volunteers first performed the fMRI experiment followed by an EEG session and vice versa. Run order of the five experimental runs was counterbalanced across volunteers.

### Data acquisition

#### EEG data acquisition

The technical details of all measurements are reported in Siemann *et al*.^9^. During training and EEG data acquisition, volunteers were seated on a height adjustable chair in front of the computer screen. Their chins rested on a chin and forehead rest (55 cm distance). In all sessions, eye movements were monitored using an in-house developed (MRI compatible) eye tracking device to assure continuous fixation at the centre of the screen throughout all experiments. EEG data were recorded from 64 channels applying the extended international 10–20 system (direct-coupled amplifier; average-reference; sampling rate: 512 Hz; impedances ≤10 kΩ). The ground electrode was placed at the left mouth angle. The electrooculogram was recorded with four additional electrodes (infra- and supraorbitally and at the outer canthi).

#### MRI data acquisition

A 3 Tesla Siemens Skyra® whole body scanner with a 20-channel head coil was used for MRI measurements. Participants wore foam earplugs and the room was dimly lit. A JVC video projector projected the stimuli that could be watched by the participants via a mirror attached to the head coil (distance to projection area: 140 cm). Participants gave manual responses using an MRI-compatible computer mouse.

Functional images were received via T2* echo-planar imaging sequences (EPI) to derive blood-oxygen level dependent (BOLD) signals (TR = 2210 ms; TE = 30 ms; flip angle = 81°; matrix = 64*64; FOV = 192*192; 41 slices; voxel size = 3 mm^3^; no gap; ascending acquisition order). Five functional runs were obtained from each individual. Each of them lasted roughly six minutes and covered 163 volumes. Anatomical scans were obtained using a T1-weighted MPRAGE sequence (TR = 1900 ms; TE = 2.07 ms; TI = 900 ms; flip angle = 9°; FOV = 256*256; 176 slices; voxel size = 1 mm^3^; roughly 4 minutes acquisition time).

To investigate brain activity during colour processing, a colour localiser scan was additionally conducted after the main experiments. For this purpose, a checkerboard was shown that covered the entire screen. The single checkerboard screens rapidly switched (8 Hz) between different colours (chromatic) or different shades of grey (achromatic) that were isoluminant with the single colours of the chromatic blocks. The chromatic checkerboard version was presented in four blocks interleaved with four achromatic blocks, each lasting approximately 20s. These were separated by baseline epochs of 10s duration in which the fixation point was shown. Data recording included 143 volumes (TR = 1800 ms; 33 slices; approx. four minutes), and a short (5 volumes) functional whole-brain scan was recorded for coregistration purposes of the functional localiser scans.

### Data analysis

#### Behavioural data

For the reaction time (RT) analysis of correct trials, a repeated-measures analysis of variance (ANOVA) was computed with the factors *method (EEG/fMRI) x cue validity (valid/neutral/invalid) x flanker congruency (CON/INC)*. Error rates (defined as percentage of incorrect trials and misses per condition) were analysed using Friedman tests. We set the significance level at α = 0.05 for all behavioural and electrophysiological analyses. In cases of the violation of the assumption of sphericity Greenhouse Geisser corrected epsilon values are reported (Mauchly’s Test). A further investigation of significant effects was carried out using post hoc paired t-tests for RTs (with Bonferroni-Holm correction where necessary) and Wilcoxon tests for error rates.

#### (f)MRI data

The functional scans were temporally resliced, coregistered to the structural scan, smoothed, and both functional and structural data were normalised to the standard MNI space. Motion parameters were estimated and used as regressors in a fixed-effects analysis using the correct trials of all six experimental conditions and the cueing period, the interstimulus, and intertrial intervals (ISI; ITI) as separate regressors. Group-specific activation of all 21 volunteers was analysed using a random-effects analysis with a full factorial design (factors: *cue validity (valid/neutral/invalid)*; *congruency (CON/INC))*. Post hoc paired t-tests were performed on the INC > CON contrast pooled over cue validity levels as well as separately for each of the cue validity levels. Moreover, conjunction analyses over the INC > CON contrasts were computed^52^, separately pooled over valid and invalid cueing, over valid and neutral cueing, and over invalid and neutral cueing to identify those regions showing congruency effects throughout different cueing conditions. In order to allow for comparisons with our previous flanker study^9^, the significance threshold for all contrasts was set to p < 0.001 (uncorrected) with an extent threshold of k ≥ 10 voxels.

Domain-general attention effects were assessed with a conjunction analysis of the flanker effects (INC > CON) over both studies (p < 0.001 uncorrected, k ≥ 10 voxels) that allowed us to identify overlapping clusters. One conjunction was computed for pooled data over all validity conditions and further conjunctions were computed separately for valid, invalid and neutral cueing, using the previously reported thresholds. To consider domain-specific attention effects the flanker contrasts (INC > CON) of both studies were directly contrasted with each other (feature > spatial and vice versa; p < 0.001 uncorrected, k ≥ 10 voxels) in order to explore possible differences between both studies.

The localiser scans were preprocessed separately. They were realigned to the 5th volume of the whole brain scan (reslice option: mean image), coregistered to the anatomical scan, (source: mean image of whole brain scan), normalised to the standard MNI space with 4th degree B-spline interpolation and resampling to 2 mm³ isotropic voxels, and smoothed with a 4 mm^3^ FWHM Gaussian kernel. A fixed-effects design was set up with separate regressors for chromatic and achromatic blocks and the fixation period. The thresholds varied between participants and hemispheres to obtain clusters of comparable sizes.

The MNI coordinates of all peaks and sub-peaks were transformed into Talairach space (‘mni2tal.m’; http://imaging.mrc-cbu.cam.ac.uk/imaging/MniTalairach). Anatomical locations were derived via the ‘Talairach Daemon Client’ software (http://http://www.talairach.org/daemon.html) and the automated anatomical labelling toolbox (‘AAL‘; http://www.gin.cnrs.fr/AAL-217?lang=en).

#### ERP data

A high-pass filter (0.05 Hz) and a notch filter (50 Hz) were used to filter the EEG data. When necessary individual channels were interpolated (spherical spline interpolation; mean = 2 channels ±2, maximum = 5 channels per participant) or defined as bad channels (mean = 0.5 channels ±1, maximum = 3 channels). Correct trials were averaged from −200 ms to 900 ms (stimulus-locked, range 70–100 trials, mean = 89 trials) per condition and participant (same number of trials in all conditions per participant).

Repeated-measures ANOVAs with the factors *cue validity (valid/neutral/invalid)* x *flanker congruency (CON/INC)* x *frontality (F/C/P/PO)* x *laterality (left/midline/right)* were carried out separately on the mean amplitudes in 5 consecutive time windows between 200–300 ms. Significant main and interaction effects were examined using post hoc paired t-tests with Bonferroni-Holm corrected thresholds.

#### fMRI-constrained source analysis

Peak coordinates were derived from fMRI contrasts of the conjunction analyses^52^ of CON and INC stimuli (pooled over validity levels) and contrasted against the intertrial interval (p < 0.005 uncorrected, k ≥ 10 voxels). The obtained 27 different peak coordinates were transformed into Talairach space (‘mni2tal.m’; http://imaging.mrc-cbu.cam.ac.uk/downloads/MNI2tal/) and clustered (minimal distance: 30 mm^53^. The maximally tolerated root mean square (RMS) distance between the derived coordinates and their primary peaks was 25 mm^54^. The resulting coordinates were used to seed regional sources (RSs) in the source analysis of the grand average ERP waveform of all participants and conditions. This source model was subsequently applied to the individual ERP data. The source model was checked using source sensitivity images derived from BESA® 6.0 (Brain Electrical Source Analysis; MEGIS Software GmbH, Munich, Germany) to evaluate potential source sensitivity problems. Finally, BCa bootstrap 95% confidence intervals were computed on the RMS of each source waveform. Only epochs of ≥20 ms were considered to reflect meaningful differences between conditions.

### Data Availability

The datasets generated and/or evaluated during the current study are available from the corresponding author on reasonable request.

## Results

### Behavioural data

Behavioural data were analysed using a repeated-measures ANOVA with the within-subject factors *method (EEG/fMRI)* x *cue validity (valid/neutral/invalid)* x *flanker congruency (CON/INC)*. All factors demonstrated significant main effects (*cue validity*: F_[2;40]_ = 7, p < 0.005; *congruency*: F_[1;20]_ = 47.8, p < 0.001; *method*: F_[1;20]_ = 71.6, p < 0.001) and the factors *congruency* and *method* interacted (F_[1;20]_ = 5.8, p < 0.05; see Table 1). Behavioural data for the fMRI session and for the EEG session are depicted in supplementary Figure S1 (fMRI session) and supplementary Figure S2 (EEG session).

**Table 1:** Behavioural data for the EEG session (left columns) and for the fMRI session (right columns; N = 21).

	Condition	EEG		fMRI	
		Congruent	Incongruent	Congruent	Incongruent
RTs [ms] ± SD	valid	511.4 ± 79.8	544.6 ± 71	577 ± 73.6	605.4 ± 63.8
	neutral	522 ± 78.9	553.5 ± 74.9	591.1 ± 75.4	616.4 ± 71.3
	invalid	530.3 ± 84	562.3 ± 83.2	598.7 ± 84.9	622.5 ± 75.8
error rates [%] ± SD	valid	3.5 ± 3	3.8 ± 3.2	3.7 ± 3	4.4 ± 2.8
	neutral	3.2 ± 2.6	4.5 ± 2.6	3.8 ± 2.5	4.6 ± 3.3
	invalid	3.6 ± 3.6	5.6 ± 4.7	8.3 ± 4.7	4.8 ± 3.9

RTs (ms), error rates (%), and respective standard deviations (SDs) are listed for all conditions.

Bonferroni-Holm corrected post hoc paired t-tests yielded that valid trials showed significantly faster RTs than invalid trials (t_[20]_ = −3.7, p < 0.005, Cohen’s d < 0.2). The *congruency* effect was characterised by higher RTs during INC trials (t_[20]_ = −6.9, p < 0.001, Cohen’s d > 0.2). RTs were generally slower during fMRI sessions than with EEG measurements (t_[20]_ = −8.5, p < 0.001, Cohen’s d > 0.8).

Analysis of the error rates using the factors *method* x *cue validity* x *congruency* yielded significant differences between conditions (Friedman test: χ_[11]_ = 39.8, p < 0.001). Post hoc Wilcoxon tests showed significantly higher error rates on invalidly cued trials compared to trials with valid or neutral cueing (Z = −3.1,p < 0.005, Cohen’s d > 1 and Z = 2.1, p < 0.05, Cohen’s d > 1 respectively), and error rates were higher during MRI measurements (Z = −2.3,p < 0.05, Cohen’s d > 1). The difference between CON and INC trials was not significant.

### fMRI data

**Figure 2:**
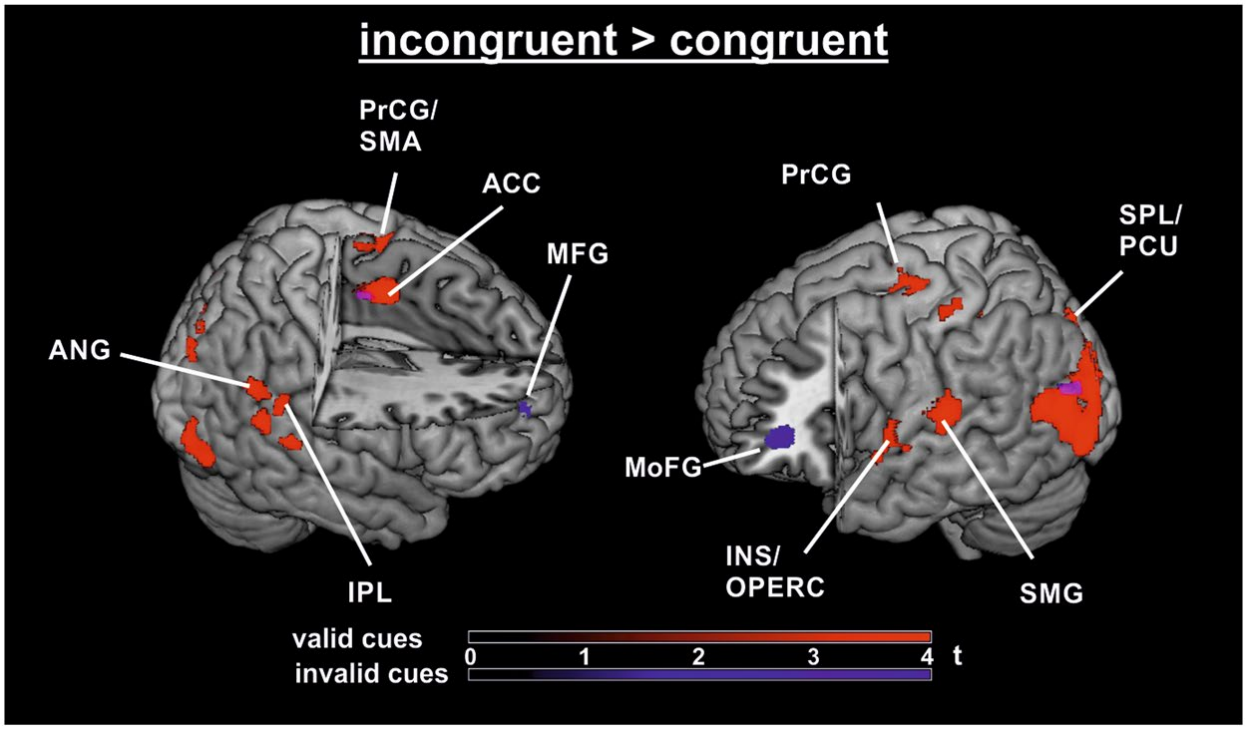
Flanker processing group fMRI activation (N = 21). Rendered activation clusters of the contrast incongruent > congruent with invalid (blue) and valid cueing (red). Bars indicate t-values (p < 0.001; k ≥ 10). ACC, anterior cingulate gyrus; ANG, angular gyrus; INS, insula; IPL, inferior parietal lobule; MFG, middle frontal gyrus; MoFG, middle orbitofrontal gyrus; OPERC, operculum; PCU, precuneus; PrCG, precentral gyrus; SMA, supplementary motor area; SMG, supramarginal gyrus.

Analysis of the fMRI data for the contrast INC > CON revealed activity in left frontal, cingulate, parietal, and occipital regions as well as right superior frontal gyrus/ supplementary motor area (SMA) and ACC. Post hoc analyses of the flanker effect (INC > CON) separately for cue validity levels yielded a broad network of activity with valid cueing (see Table 2 and Fig. 2). Structures bilaterally activated included inferior parietal lobule (IPL), supramarginal gyrus (SMG), lingual gyri (superior occipital gyrus /middle occipital gyrus), and ACC/SMA. In the left hemisphere, there was also activation in superior and middle frontal gyri (MFG), superior parietal lobule (SPL), and insular cortex. Right hemispheric clusters were significant in angular gyrus, superior temporal gyrus, and inferior occipital cortex. The congruency effect with invalid cueing was associated with an activation pattern in bilateral orbitofrontal cortices, left middle occipital gyrus, middle cingulate gyrus/SMA, and right superior frontal gyrus. With neutral cueing, only the bilateral caudate bodies showed suprathreshold activation, and separate conjunction analyses over neutral and valid and neutral with invalid INC > CON contrasts yielded no significant clusters.

**Table 2:** Activated clusters for the contrast incongruent > congruent (INC > CON) pooled over cue validities (above; N = 21).

Contrast	Cluster size	t-Value	r	Peak coordinates (Talairach)			Hemisphere	Anatomical Region [BA]
INC > CON (all cue types)	1216	4.9	5	−42	−73	20	L	Middle temporal gyrus^39^
		4.8	4	−40	−80	2	L	Middle occipital gyrus^19^
		4.7	0	−30	−82	21	L	Middle occipital gyrus^19^
	771	4.6	0	−30	−7	56	L	Middle frontal gyrus^6^
		4.1	0	−22	−12	63	L	Precentral gyrus^6^
		3.9	2	−48	−21	54	L	Postcentral gyrus^1^
	357	4.4	0	−4	−2	42	L	Cingulate gyrus^24^
		3.9	4	−12	0	33	L	Cingulate gyrus^24^
		3.9	3	−10	−23	45	L	Cingulate gyrus^31^
	26	4.1	1	−53	4	33	L	Precentral gyrus^6^
	19	3.8	0	−26	−52	−24	L	Culmen
	47	3.7	2	0	47	9	L/R	Anterior cingulate^32^
	58	3.7	1	−18	−57	60	L	Superior parietal lobule^7^
	18	3.7	3	28	55	8	R	Superior frontal gyrus^10^
	23	3.5	0	26	−48	−21	R	Culmen
		3.4	0	26	−57	−17	R	Declive
	10	3.4	2	18	3	61	R	Medial frontal gyrus^6^
INC > CON (valid & invalid cues)	20	3.7	5	−42	−73	20	L	Middle temporal gyrus^39^
	14	3.3	0	−2	−13	43	L	Paracentral lobule^31^

Significant clusters for the conjunction of valid and invalid trials are shown below (p < 0.001 uncorrected; k ≥ 10); BA = Brodmann area; L = left hemisphere; R = right hemisphere; r = range of nearest gray matter.

By contrast, a conjunction analysis^52^ over the validly and invalidly cued INC > CON contrasts yielded significant clusters showing congruency effects in both cueing conditions (p < 0.001; k ≥ 10). Suprathreshold activation clusters were evident in left middle occipital gyrus and middle cingulate gyrus/SMA with a peak in paracentral lobule.

With respect to domain-general attention effects, a conjunction for the INC > CON contrast over spatial and feature-based data (and pulled together over validities) yielded small clusters in left cuneus (13 voxels, x/y/z Talairach peak coordinates: −26/−78/26) and in left MFG (19 voxels, −24/−12/56).

The same conjunction (over spatial and feature-based attention) for the flanker effect was also computed separately for all three validity levels. Only the conjunction for valid cueing revealed significant clusters in left precuneus (33 voxels, x/y/z Talairach coordinates −26/−74/20) and in left fusiform gyrus (17 voxels, −40/−70/−10). Corresponding conjunction contrasts for the flanker effect with invalid and also with neutral cueing did not result in suprathreshold clusters, probably as there were no clusters in the spatial cueing experiment for these contrasts.

Domain-specific clusters were extracted by contrasting both attention types (spatial > feature-based and vice versa) with each other with respect to flanker processing pooled over validity levels (INC > CON contrast). Whereas spatial attention compared to feature-based attention yielded no suprathreshold clusters, several brain areas showed higher activity in the feature-based experiment during flanker processing, including left middle occipital gyrus and bilateral middle temporal gyrus.

### ERP data

To analyse the N200 component, mean amplitudes of INC and CON conditions separately for all cue validity levels were examined between 200–300 ms using separate repeated-measures ANOVAs with the factors *frontality (F/C/P/PO)* x *laterality (left/midline/right)* x *cue validity (valid/neutral/invalid)* x *flanker congruency (CON/INC)* in consecutive time bins (each 20 ms).

**Figure 3:**
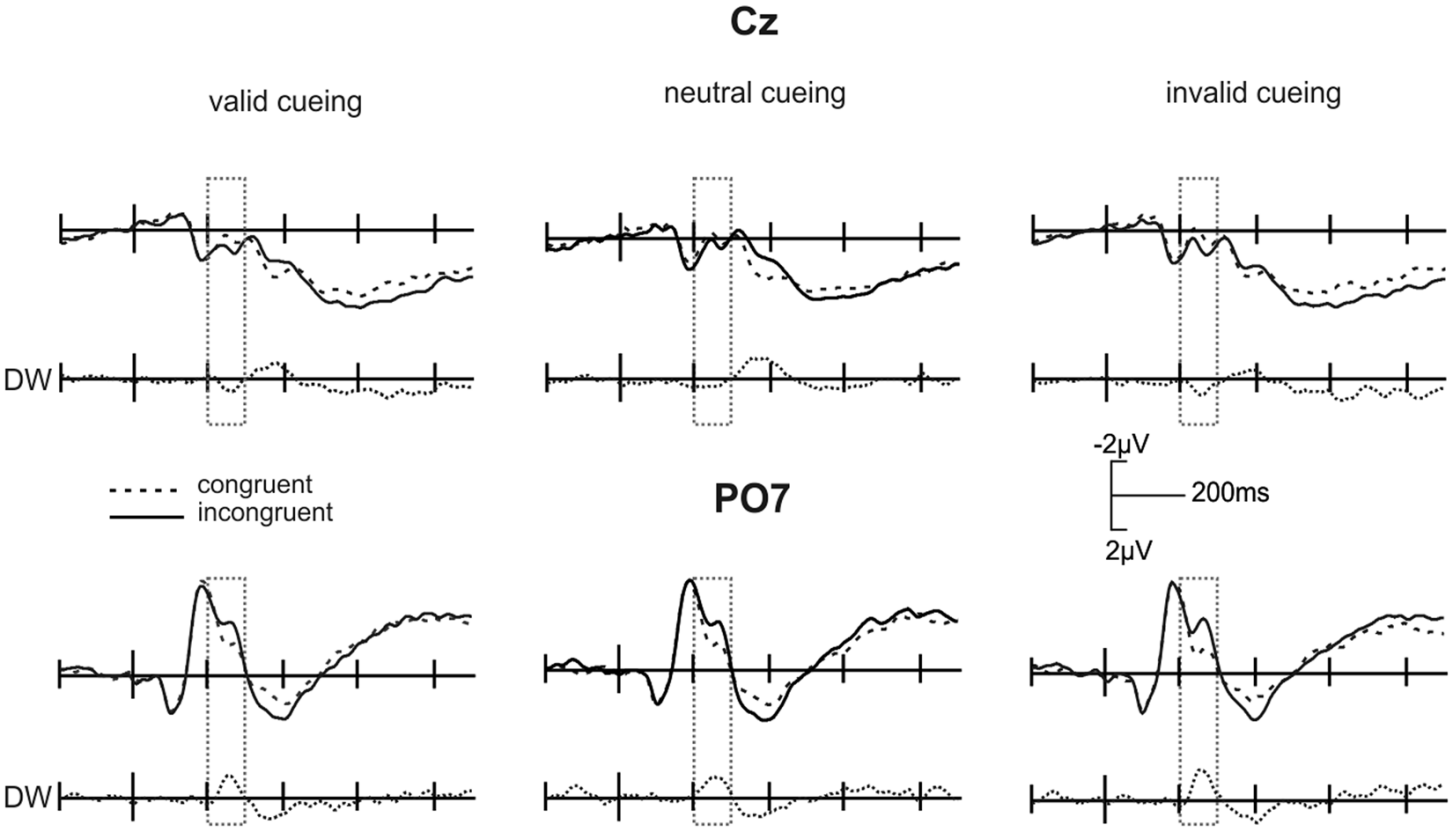
Grand average ERPs and difference waves (DW) for flanker interference (stimulus-locked: –200 to 900 ms). Congruent and incongruent conditions are shown with valid (left), neutral (middle) and invalid (right) cueing at electrode positions Cz (top) and PO7 (bottom). Difference waves are denoted below the grand averages (N = 21).

In general, the difference waves (INC > CON) were characterised by an early relative negativity for INC trials at bilateral parieto-occipital electrodes and a simultaneous fronto-central relative positivity. From 200 ms–220 ms, the factor *congruency* demonstrated no main effect or any interaction effect with the other factors. In the time windows from 220 ms–240 ms, 240–260 ms, and 260–280 ms, *congruency* interacted with both *frontality* (F_[1.3;26.2]_ = 8.2, p < 0.01, F_[1.3;26.5]_ = 20.8, p < 0.001, and F_[1.4;27.6]_ = 12.8; p < 0.001 respectively) and *laterality* (F_[2;40]_ = 3.8, p < 0.05, F_[2;40]_ = 8.3, p < 0.005, and F_[2;40]_ = 3.8; p < 0.05 respectively). Post hoc t-tests between 220–240 ms yielded significant congruency effects at electrodes Cz (t_[20]_ = −3.5; p < 0.005), C4 (t_[20]_ = −3.2; p < 0.01) and PO7 (t_[20]_ = 4.0; p < 0.005). ERPs at electrode positions Cz and PO7 are depicted in Fig. 3.

From 240–260 ms post hoc tests showed significant differences between congruency conditions at electrodes Fz (t_[20]_ = −3.5; p < 0.005), F4 (t_[20]_ = −4.8; p < 0.001), C3 (t_[20]_ = −3.3; p < 0.005), Cz (t_[20]_ = −4.7; p < 0.001), C4 (t_[20]_ = −4.5; p < 0.001), P3 (t_[20]_ = 3.4; p < 0.005), P4 (t_[20]_ = 3.2; p < 0.01), PO7 (t_[20]_ = 7.0; p < 0.001) and PO8 (t_[20]_ = 3.8; p < 0.005). In the time window from 260–280 ms, post hoc tests demonstrated a *congruency* effect at electrodes F4 (t_[20]_ = −3.4; p < 0.005), P4 (t_[20]_ = 2.9; p < 0.01), PO7 (t_[20]_ = 5.6; p < 0.001) and PO8 (t_[20]_ = 3.3; p < 0.005).

In the last window from 280–300 ms, there was a three-way interaction of flanker *congruency*, *validity* and *laterality* (F_[4;80]_ = 3.1; p < 0.05). A post hoc t-test yielded only sub-threshold differences between the single conditions at left, middle and right electrode sites.

### fMRI-constrained source analysis

#### Clustering of fMRI peak coordinates

RSs were derived from clustering of the fMRI peak coordinates (in Talairach space) analogous to Siemann *et al*.^9^ and described in more detail in Bledowski and colleagues^55^. The resulting 10 RSs were located in left precentral gyri (two RSs), parietal lobe, cerebellum, and ACC, as well as in right postcentral gyrus, cerebellum, MFG, precuneus, and ACC (see Table 3).

**Table 3:** Coordinates of the resulting regional sources and the original peak coordinates from fMRI (N = 21).

Conj	Region [BA]	r	Peak coordinates (Talairach)			RS Region [BA]	RS coordinates (Talairach)		
			x	y	z		x	y	z
C/I	Inferior frontal gyrus^9^	2	−57	7	31	Precentral gyrus^6^	−56	−5	39
C	Postcentral gyrus^1^	0	−55	−17	44				
I	Postcentral gyrus^1^	1	−44	−18	58				
C/I	Precentral gyrus^6^	0	−32	−16	65	Precentral gyrus^6^	−33	−12	62
C	Middle frontal gyrus^6^	0	−30	−5	61				
I	Superior frontal gyrus^6^	0	−26	−7	63				
C	Culmen	0	−38	−56	−26				
C	Culmen	0	−38	−44	−30	*Culmen*	−*32*	−*44*	−*28*
C	*	0	−26	−38	−28				
I	*	*	−32	−58	8		−27	−50	22
C/I	Parahippocampal gyrus^30^	4	−30	−50	4				
C	Angular gyrus^39^	6	−28	−53	34	WM (Parietal Lobe)			
I	Caudate tail	4	−20	−40	15				
C	Caudate tail	5	−18	−40	15				
C/I	Anterior cingulate cortex^24^	0	−4	2	39	Anterior cingulate cortex^24^	−4	2	39
C/I	Postcentral gyrus^43^	4	57	−16	21	Postcentral gyrus^43^	57	−16	21
C/I	Culmen	0	38	−56	−24				
C/I	Culmen	0	34	−42	−30	*Culmen*	33	−*47*	−*18*
I	Hippocampus	2	32	−41	0				
C/I	Culmen	0	28	−48	−19				
I	Middle frontal gyrus^6^	0	30	−3	61	Middle frontal gyrus^6^	30	−3	61
C/I	Precuneus^7^	0	16	−59	55	Precuneus^7^	16	−59	55
C/I	Medial frontal gyrus^10^	2	20	45	11				
I	Inferior frontal gyrus^47^	5	20	35	−2	Anterior cingulate cortex^24^	10	31	4
C/I	Anterior cingulate cortex^24^	4	4	23	1				
I	Caudate body	1	−6	20	6				
RS seeded (coordinates based on colour localiser)						Lingual gyrus^18^	−28	−73	−9
RS seeded (coordinates based on colour localiser)						Lingual gyrus^18^	29	−70	−7

On the left, peak coordinates are listed for the conjunction contrasts of all congruent conditions > fixation (C) and all incongruent conditions >fixation (I), (p < 0.005, uncorrected, k ≥ 10). Resulting regional source coordinates after clustering are shown on the right with corresponding anatomical labels and Brodmann areas (BA). All data refer to Talairach coordinates. RSs in italics were excluded from the final source model. Conj = conjunction; r = range of nearest gray matter; WM = white matter.

One of the precentral gyrus sources was close (RMS distance: 17.6 mm) to a RS derived in our spatial cueing study, where it seemed to reflect activation in left IFG. Due to the similarity of these sources between experiments and given that the source in the present study was based on peak coordinates derived from activity clusters in left IFG and in postcentral gyrus, this precentral gyrus RS is henceforth considered to reflect source activity originating in left IFG. Furthermore, in line with two of our previous studies applying source analysis^9,53^ the cerebellar sources were excluded from further analyses. Each of them only explained less than 1% of the overall variance.

A principle component analysis (PCA) was carried out on the residual signal remaining after seeding of the eight fMRI-constrained RSs. This PCA revealed two principle components that explained 8.1% and 1.4% of the residual variance between 50 ms and 150 ms. These components were assumed to reflect perceptual processing of visual stimulus features. Due to the cueing manipulation of the stimulus colour, two RSs seeds were based on the mean of the individual peak coordinates found in the additionally conducted colour localiser scans (see Table 4) that best matched the coordinates of colour-sensitive regions (MNI coordinates: ±32/−82/−29; according to^56^). The colour localiser derived brain activity was located in bilateral lingual gyri and was in close spatial proximity to two additional RSs that were fitted in Siemann *et al*.^9^ (RMS distance: 4.6 mm and 7.6 mm for left and right RSs respectively). The resulting source model was saturated based on principle component analysis of the residual variance (<1% residual variance of all components) with a Goodness of Fit (=100% minus residual variance) of 97.9% ± 0.9%.

**Table 4:** Individual MNI peak coordinates resulting from the chromatic > achromatic contrast of the checkerboard colour localiser using individually defined thresholds for the left and right hemisphere respectively.

Participant	Cluster size	Left hemisphere (MNI space)				Right hemisphere (MNI space)				
		x	y	z	Distance (mm)	Cluster size	x	y	z	Distance (mm)
**01**	32	−20	−80	−18	12.3	38	28	−78	−8	13.3
**02**	30	−28	−80	−2	18.5	35	22	−72	−14	15.4
**03**	31	−22	−78	−14	12.3	36	28	−78	−12	9.8
**04**	34	−24	−68	−14	17.2	37	28	−72	−16	11.5
**05**	sub	−34	−78	−18,0	4.9	33	26	−64	−12	20.6
**06**	32	−20	−80	−14	13.6	33	30	−74	−16	9.2
**07**	33	−24	−82	−14	10.0	24	30	−72	−8	15.7
**08**	35	−34	−76	−18	6.6	33	26	−80	−14	8.7
**09**	32	−30	−70	−14	13.6	30	34	−68	−18	14.3
**10**	NaN	NaN	NaN	NaN	NaN	NaN	NaN	NaN	NaN	NaN
**11**	36	−30	−68	−14	15.4	33	36	−64	−14	19.4
**12**	30	−22	−74	−16	13.4	31	30	−78	−16	6.0
**13**	31	−32	−80	−14	6.3	33	26	−82	−16	7.2
**14**	30	−30	−80	−18	3.5	30	32	−68	−16	14.6
**15**	37	−28	−74	−16	9.8	30	30	−72	−12	13.0
**16**	30	−36	−76	−18	7.5	32	30	−74	−2	19.8
**17**	40	−26	−68	−10	18.2	30	26	−70	−14	14.7
**18**	36	−34	−68	−10	17.3	35	32	−80	−12	8.2
**19**	38	−32	−66	−16	16.5	37	30	−66	−14	17.2
**20**	31	−26	−72	−14	13.1	39	26	−70	−14	14.7
**21**	29	−32	−72	−18	10.2	28	26	−66	−12	18.9
**Group**		−28	−75	−15	10		29	−72	−13	12.3

Distances were computed between each peak coordinate and reference coordinates reported in the literature (see Luna *et al*., 1998). Bottom: Mean coordinates over all participants used as seed coordinates (transformed into Talairach space) for the source analysis. NaN = definition of colour-sensitive region not possible; sub = sub-peak.

#### Source waveform data

**Figure 4:**
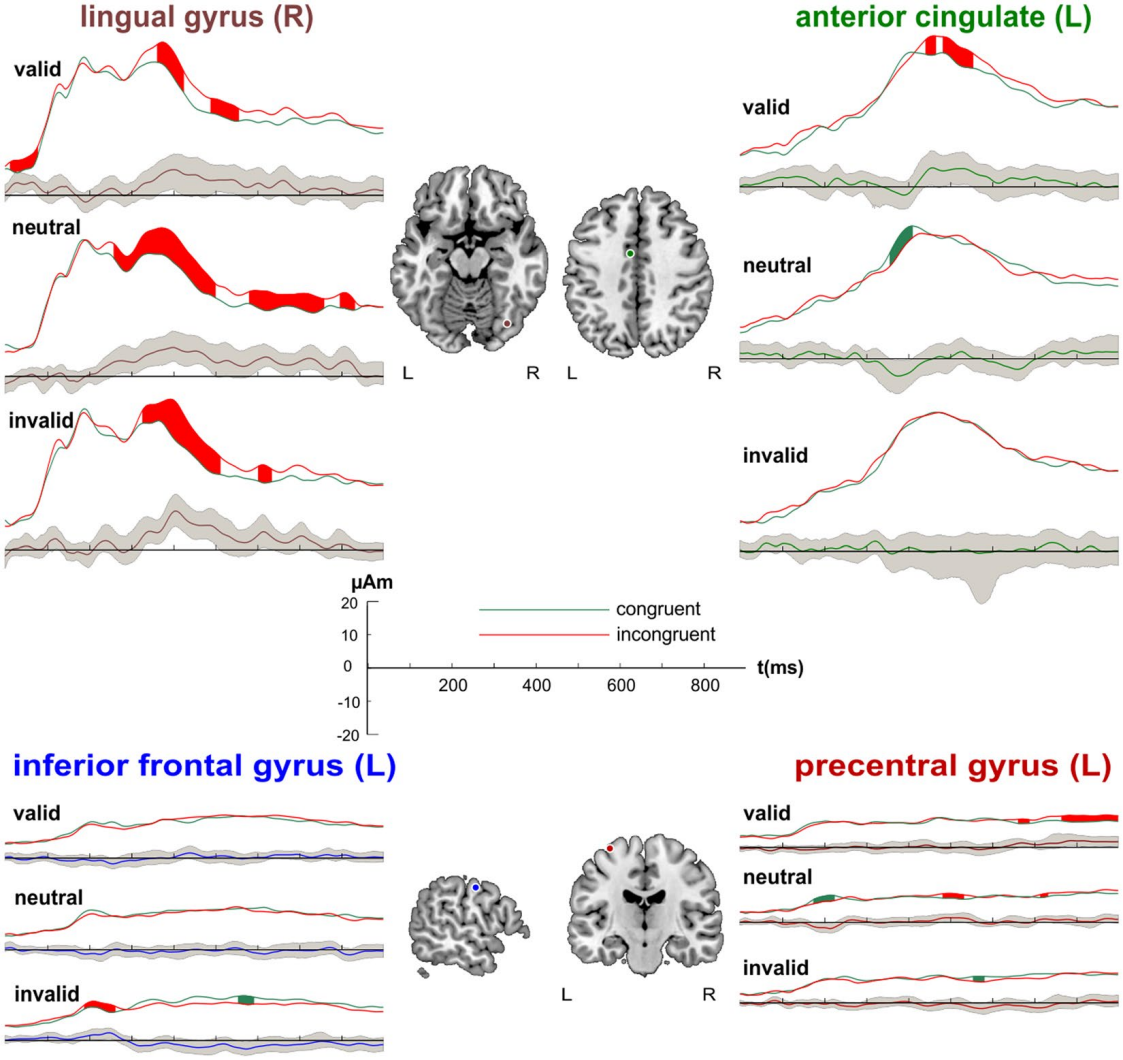
Locations and source waveforms of regional sources for flanker interference (N = 21). Middle: locations of the regional sources in right lingual gyrus, left anterior cingulate gyrus, left inferior frontal gyrus, and left precentral gyrus projected onto the Talairach-transformed MNI brain. Root mean square source waveforms are shown for each regional source and condition (0–900 ms). Congruent conditions are presented in green, incongruent conditions in red. Below the source waveforms difference waves are shown in the respective colour of the regional source. The BCa bootstrap 95%-confidence interval is depicted as a grey area surrounding the difference waves. Epochs deviating from zero for > 20 ms are considered significant and coloured in green (congruent > incongruent) and red (incongruent > congruent). L = left hemisphere; R = right hemisphere.

Within each cue validity level, source waveforms of INC and CON trials were statistically compared using BCa bootstrap 95%-confidence intervals^57^. The positions of RSs with significant effects and their source waveforms per condition are shown in Fig. 4. The corresponding time windows with significant effects are listed in Table 5.

**Table 5:** Time epochs (in ms) with significant BCa bootstrap 95%-confidence intervals where the difference between incongruent and congruent Flanker conditions did not include zero (N = 21).

Regional Source	valid cueing [ms]	neutral cueing [ms]	invalid cueing [ms]
left inferior frontal gyrus			**183–261**
			553**–**589
right postcentral gyrus	**69–89**		**0–30**
left anterior cingulate gyrus	**439–466**	355**–**408	**96–164**
	**479–553**		
left precentral gyrus	**661–689**	172**–**225	553**–**580
	**763–900**	**476–533**	
		**712–734**	
left inferior parietal lobule	**4–50**	**563–600**	**228–260**
	**84–111**	853**–**900	291**–**329
	**446–516**		**555–711**
	**534–576**		**718–757**
	**582–617**		
	**622–670**		
right middle frontal gyrus	**475–540**	300**–**326	**168–200**
	**618–664**	**470–581**	**797–857**
right precuneus	371**–**417	126**–**148	300**–**427
	**500–603**	343**–**467	433**–**474
	**632–667**		**600–680**
			**711–764**
			**815–845**
right anterior cingulate gyrus	**469–507**	**26–67**	
	**517–684**	**93–130**	
	**690–719**	**800–825**	
left lingual gyrus	**348–369**		**500–644**
			**670–784**
			**862–900**
right lingual gyrus	**11–76**	**256–500**	**325–510**
	**360–423**	**580–757**	**601–635**
	**487–554**	**796–830**	

Data are shown separately for each validity level for time epochs ≥20 ms (left = valid cueing; middle = neutral cueing; right = invalid cueing). Time epochs with higher source activities for incongruent than congruent conditions are indicated in bold face. The remaining epochs show the reverse direction.

The RS in left IFG showed early congruency effects with invalid cueing overlapping in time with the N200 time window and differences during later stimulus processing. There were no significant epochs for the other cueing conditions. The RS in right postcentral gyrus demonstrated early differences between INC and CON when cues were neutral. Additionally, there were source waveform differences arising in bilateral ACC and precentral gyrus with valid and neutral cueing in later phases of the analysed time window. Invalidly cued trials yielded late differences in source activity between INC and CON stimuli in precentral gyrus and right ACC. Furthermore, frontal and parietal RSs were sensitive to stimulus congruency during early stimulus presentation with valid (inferior parietal lobule) and invalid (MFG; inferior parietal lobule) cueing and during later phases with all cue validity types. The postcentral gyrus RS demonstrated early congruency effects when cues were valid or invalid. The precuneus source yielded early congruency effects with neutral cueing and late effects regardless of cue validity level. Finally, the sources in the lingual gyri contributed to source waveform differences between INC and CON stimuli during early stimulus processing (valid and neutral cueing) and at later phases (valid and invalid cueing).

## Discussion

### Feature-based attentional selection mechanisms

#### Behavioural data

In the behavioural data analysis, significant RT effects were evident for the factors *flanker congruency* (CON < INC), *cue validity* (valid < invalid), and *method* (EEG < fMRI), and more errors were committed on invalid compared to valid and neutral trials. Most importantly, there was no interaction of cue validity and flanker congruency, which has been shown for spatial cueing experiments using flanker-like tasks in previous studies^32–35^. Past accounts on feature-based attention postulate that spatial and feature-based attention types differ with regard to underlying mechanisms and temporal patterns^10,58^. Based on these theories, a beneficial effect of correctly attending to the stimulus colour cannot be expected. However, the guided search model^11^ assumes that early selection based on features is indeed possible.

Obviously, our results are more in line with feature integration theory^10^, which might result from the choice of experimental parameters in the present study. Accordingly, a factor that may have interfered with early selection could be the unpredictable stimulus onset due to a jittered interstimulus interval between cues and stimuli. Abrupt target onsets are more likely to capture attention than salient information alone^59^. Moreover, in a study contrasting interference processing with valid spatial attentional orienting, beneficial effects were only visible for exogenously driven reorienting (phasic alertness). That same study found no reduced interference effects for endogenous attentional shifting^60^. Therefore, by using an unpredictable stimulus presentation timing (jittered ITI) together with a sudden onset, we may have introduced early attentional captures to the target location. This may have interrupted a global feature-based search mode, promoting focussing mechanisms which interfered with a global search mode generally associated with feature-based experiment^61,62^.

Moreover, the experimental manipulations accounted for a small amount of the variation of RTs between participants (small effect sizes), whereas the factor *method* contributed substantially (large effect size). Therefore, the observed two-way-interaction of *congruency* x *method* was probably driven by differences in the response patterns between the experimental environments (EEG vs MRI). Responses were generally slower in the MR scanner than in the EEG experiment, which is in line with previous studies (e.g.^63,64^), but rarely discussed. Koch and colleagues^65^ examined RT differences for task switching between common behavioural, real, and simulated MRI settings. They attribute the slower responses in the MRI setting to distracting factors (monotonous noise, horizontal position, see also ref.^66^) causing a slowdown in late motor processes. Likewise, the error rates also demonstrated large differences (Cohen’s d > 1) between methods, especially for invalid conditions. Higher error rates in the invalid condition were an expected pattern, possibly further augmented by distracting factors also affecting RTs as previously described.

#### Neurophysiological data

The dynamics of flanker interference processing observed in the present study differed between validity levels. Hence, we will first discuss mechanisms leading to activity in fronto-parietal structures on validly cued trials. Afterwards, we will address processing mechanisms we postulate during invalid trials. Neutral trials will be discussed within both parts where appropriate.

In the present experiment, flanker conflict (INC > CON) with valid colour cues led to significant BOLD signal increases in a broad network mainly comprising parietal lobules (superior/inferior), as well as MFG. The joint involvement of fronto-parietal structures hints at an interaction of top-down and bottom-up processes: Concerning top-down control, SPL and MFG may represent domain-independent cortical regions that are active in response to execution attentional shifts. Both were reported in conditions requiring updating of an attentional goal^36^. In the present study, we assume that frontal regions activated attentional orienting mechanisms. Thus, the peak coordinates of the activated MFG cluster are close to the putative frontal eye field (FEF; x/y/z Talairach peak coordinates: −30/−4/49 according to^67^; RMS distance: 8 mm). FEF forms part of the dorsal fronto-parietal network^13^ and is involved in trial-by-trial top-down control processing to adjust the attentional focus^68^. This is possibly implemented through connections to visual cortical regions^69^ to establish and continuously update a priority map coding the saliency of each location^70^. Enhanced activity in FEF on validly cued INC trials is therefore consistent with the interpretation of the behavioural response pattern. It delivers neurophysiological evidence that spatial attentional mechanisms were involved due to attentional capture despite an initial feature-based search mode^38^. Furthermore, the INC > CON cluster with a peak in right superior temporal gyrus (x/y/z: 59/−46/19) overlaps with posterior temporoparietal junction (TPJ), as indicated by close proximity to TPJ locations reported in the literature^71^ (x/y/z of mean right TPJ Talairach peak coordinates: 59/−48/19; RMS distance: 2 mm). A recent neurocognitive framework of attention^72^ assumes that visual cortical regions such as V4 communicate with both the ventral (TPJ) and the dorsal (SPL) fronto-parietal network during attentional orienting. Given the concurrent clusters in left SPL and FEF, the observed fMRI patterns points towards a simultaneous involvement of two fronto-parietal orienting networks in flanker conflict processing with valid cueing. TPJ of the ventral fronto-parietal network potentially received visual input about the stimulus colour and shape (ventral visual stream^73^), while the dorsal fronto-parietal network (SPL; FEF) probably processed spatial information (dorsal visual stream^74^). TPJ activity is generally considered to reflect bottom-up driven orienting reactions to salient and target-related information at unattended locations. Thus, disrupting TPJ activity via TMS interferes with conflict processing, specifically with stimulus-based flanker but not response-based Simon conflict^75^, fostering the idea that TPJ activity is essential for bottom-up attention. Increased connectivity patterns from TPJ to FEF reported in the literature^76,77^ are considered to show that ventral attention regions signal violations of expectations to the dorsal network, which in turn executes reorienting mechanisms^78^.

Further clusters that were evident in the INC > CON contrast with valid feature-based cueing were located in left-hemispheric precentral gyrus and insula/operculum, right superior temporal gyrus, and in bilateral inferior parietal lobule and bilateral cingulate gyri. This broad activation pattern implies that attending to features requires a large network of structures to overcome conflict in addition to the aforementioned fronto-parietal control structures. The left ACC cluster was part of the rostral cingulate zone, which plays a role in response selection in ambiguous situations more generally^79^. Therefore, it might reflect increased difficulties regarding response selection or execution. Correspondingly, left ACC also showed a significant cluster in a conjunction analysis of INC > CON with both valid and invalid feature-based cueing, pointing towards similar conflict resolution mechanisms independent of cue validity. The concurrent cluster in precentral gyrus also suggests that response generation was more difficult or prolonged on INC compared to CON trials. Similarly, effective connectivity from ACC towards precentral gyrus on valid trials is enhanced during conflict trials, hinting at a role for ACC in facilitating response selection or execution^80^.

Source waveform data of RSs (overlapping anatomically with the fMRI clusters) in ACC and precentral gyrus corroborate the assumption outlined above. Thus, differences between INC and CON trials started earlier in the RS in ACC (~440 ms) than in precentral gyrus (~660 ms) with valid cueing. As the congruency effect in ACC occurred relatively late during stimulus processing, it may reflect conflict resolution, whereas precentral gyrus probably demonstrated enhanced source activities due to competing responses on INC trials. ACC signals were possibly transmitted to precentral gyrus in order to facilitate response selection^80^.

Further comparing valid and invalid trials (conjunction analysis of INC > CON contrasts), the observed fMRI clusters for the INC > CON contrast overlapped in middle occipital and cingulate gyri but were otherwise distinct (orbital and superior frontal gyri on invalid trials). This seems to indicate that SPL and MFG were not significantly involved in flanker processing on invalid trials. Alternatively, we suggest that invalidly cued trials may generally have activated both SPL and MFG^13^, leading to comparable activation levels of SPL and MFG on CON and INC trials. In turn, a ceiling effect of activity in SPL and MFG may have shadowed additional activity increases on invalid INC compared to invalid CON flanker trials.

The overlapping cluster in bilateral occipital (including lingual) gyri hints at a generally enhanced recruitment of visual regions when features are attended. In line with this suggestion, feature-based attention was found to enhance activity in visual regions processing the attended feature dimension^81^. Hence, there is stronger activation in response to distracters in target colours than those in irrelevant colours in visual regions that retinotopically correspond to distracter locations^70^. Activity in occipital regions in response to conflict may thus result from attentional captures. The target stimuli were always presented in one of the task-relevant colours, possibly inducing higher levels of saliency^13,82^. Moreover, the distracters appeared in the same colour as the target letter and may generally have captured attention, especially if colour was attended to (valid and invalid trials). As a complementing finding, the present ERP data demonstrated a parieto-occipital negative voltage difference between INC and CON trials within the N200 time window. The posterior negativity may originate in occipital brain regions, as there were corresponding source waveform differences observable in RSs in lingual gyri. Accordingly, lingual gyrus is part of colour-sensitive visual area V4^83,84^ and seems to be sensitive to both spatial and feature-based attention^85^.

### Domain-general and domain-specific attentional mechanisms

In the following, we will embed the findings and interpretations of the present study into the information available from our previous study with spatial cueing. First, the pattern of behavioural results will be compared. Subsequently, we will address evidence hinting at a domain-general network involved in both experiments as well as neurophysiological mechanisms that we found to be complementary in the sense of domain-specific processes.

### Behavioural data

The behavioural size of the overall flanker effects (INC > CON) was comparable across our two cueing experiments (mean ± SD: spatial cueing: 40 ± 18 ms, Cohen’s d > 0.5 feature-based cueing: 29 ± 19 ms, Cohen’s d > 0.2). Neither of the studies showed interactions between cue validity and flanker congruency. The similarity between the results patterns of our two studies corroborates our interpretation outlined above that attentional focussing mechanisms were involved: As stimulus onsets were unpredictable in both studies, overlapping mechanisms between experiments with respect to orienting reactions are likely. However, contrary to our previous experiment with spatial cues, other studies found spatial cueing to reduce interference effects. While this was not to be expected in the present feature-cueing study based on feature integration theory^10,11^, it is of particular note that both studies yielded comparable results with respect to the size of the flanker effects (especially on validly cued trials). We assume that shifts on invalidly cued trials in the spatial cueing study were always implicitly required as attention switched from the cued to the target location. This is contrary to invalid trials in the feature-based cueing study, where the search mode was initially not focussed on a specific location, so that changing the mode from global to focussed instead of reorienting in space was probably involved. In the spatial experiment, this additional attentional reorienting may have provided sufficient time to identify the flanker conflict so that beneficial effects of validly directed attention (i.e. reduced interference effects) might have been shadowed when compared to invalid trials. That is, we believe that valid spatial cueing indeed reduced flanker interference processing compared to invalid cueing, but the flanker effect was partly processed during reorienting on invalid trials. In the attention network test, similar mechanisms could be shown for spatial shifts of attention^86^.

The same line of argumentation may apply to violations of feature-based attention in the present study irrespective of cue validity: Abrupt stimulus onsets potentially captured attention on all trials similar to invalidly cued trials in the spatial cueing experiment, and when the stimulus colour additionally deviated from the expected colour (invalid cueing) recalibration mechanisms probably became necessary^36^. Therefore, similar to spatial shifting between locations in Siemann *et al*.,^9^ the time required to update the attentional task set potentially sufficed for conflict resolution in the present study. This might have led to comparable flanker effect sizes on valid and invalid trials. A recent study manipulated attentional capture effects on feature-based attention^87^ and found spatial filtering to be more effective than onset effects, supporting the idea that interference effects are not obligatory (see e.g.^34^). However, they used 100% cue validity in order to prevent globally directed or divided attention. Both mechanisms (global/divided attention) were probably involved in our studies (feature-based/spatial cueing). Ruthruff *et al*.’s study^87^ therefore supports our argument of attentional captures contributing to overlapping results patterns across our studies.

In sum, the similarity between studies may derive from attentional switch mechanisms evident in both experiments. First, invalid spatial cueing probably led to attentional shifts. These shifts might have masked longer response selection times on INC trials compared to validly cued INC stimuli because the conflict could be processed during this assumed shift. Second, using feature-based cues, attentional captures potentially interrupted a global feature-based search mode on both valid and invalid trials. Likewise, Andersen *et al*.^88^ found that local objects sharing the target colour interfere with global feature-based attention, effectively constraining it. Furthermore, unexpected colour presentations on invalid trials yielded additional attentional recalibration mechanisms, thereby masking the additional time required for flanker conflict processing on invalid compared to valid trials.

### Neurophysiological data

With respect to neurophysiological activity, brain regions involved in flanker conflict processing partly overlapped between both experiments. In addition, several RSs that were evident in both source models were analogous, suggesting that similar networks were recruited with both attention types. In the following, we will first discuss similarities between experiments (putative domain-general mechanisms) and subsequently the observed differences including distinct temporal dynamics (putative domain-specific mechanisms).

### Domain-general network

**Figure 5:**
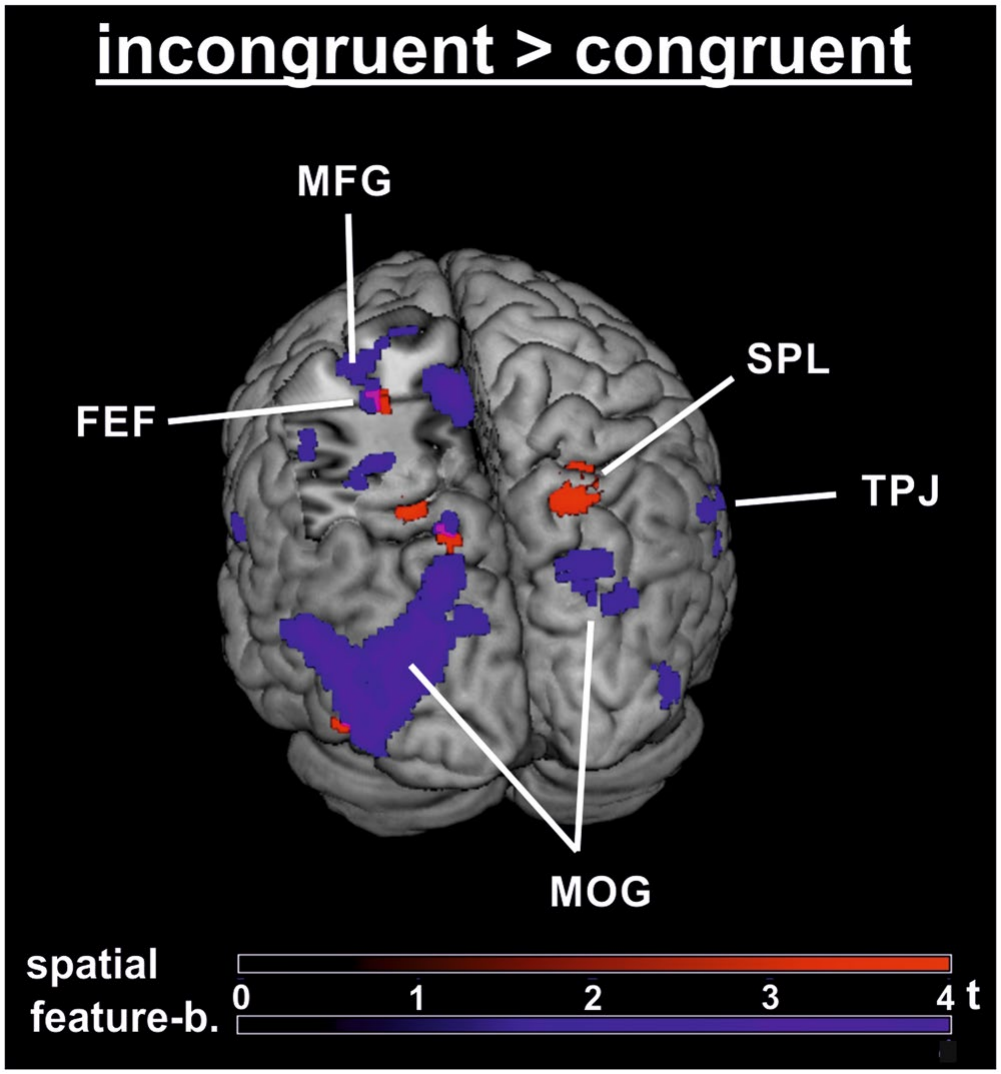
Group fMRI activation for flanker interference with spatial and feature-based cueing. Rendered activation clusters of the contrast incongruent > congruent with spatial (red; N = 19) and feature-based cueing (blue; N = 21). Bars indicate t-values (p < 0.001; k ≥ 10). ACC, anterior cingulate gyrus; FEF, frontal eye field; MFG, middle frontal gyrus; MOG, middle occipital gyrus; PCU, precuneus; SPL, superior parietal lobule; TPJ, temporoparietal junction.

Left SPL and MFG were active in the main fMRI contrast INC > CON with spatial cueing (x/y/z Talairach peak coordinates: SPL: −24/−56/49; MFG: −26/−8/41) as well as with feature-based cueing (SPL: −18/−57/60; MFG: −30/−7/56). These clusters were also evident in the subset of validly cued trials, where the same regions showed significant activation clusters in the spatial (SPL: −24/−58/53; MFG: −24/−4/43) and the feature-based cueing experiment (SPL: −14/−63/51; MFG: −26/−2/44). Activated clusters in the main contrast for INC > CON for features-based and for spatial cueing are depicted in Fig. 5. An analogous MFG cluster was also visible in a conjunction analysis over both experiments pooled over validities (MFG: −24/−12/56). By contrast, whereas the feature-based cueing experiment yielded suprathreshold clusters of the INC > CON contrast with invalid as well as neutral cueing, the same contrasts did not lead to any significant clusters in the spatial experiment. This finding explains lacking activation in the conjunction analyses over both experiments for invalid or neutral cueing.

Comparing valid cueing between studies, MFG seems to be the most consistently activated region. We may thus conclude that MFG (or FEF, as we denote the cluster based on the literature) is specific to conflict processing mechanisms under the influence of top-down control on valid trials. It responds to correctly directed attention with regard to both location and colour and shows enhanced activity during interference (INC > CON). In addition, putative FEF was also involved in flanker conflict processing in the spatial cueing experiment (x/y/z Talairach peak coordinates: −30/−4/49 according to^67^; RMS distance: 10 mm). Considering similar SPL and FEF activation between both of our cueing studies, it is likely that valid cueing initiated comparable attentional control mechanisms with location and colour cues. Together with SPL, MFG belongs to the dorsal fronto-parietal network^13^ for top-down attentional orienting processes which is active during endogenous attentional cueing^14^. The results are therefore compatible with our a priori hypothesis of overlapping activity in dorsal fronto-parietal regions. However, it is also conceivable that MFG is more generally involved in focusing attention, given that temporarily disrupting MFG had no deteriorating effect on different conflict manipulations in a recent TMS study^89^.

Actually, due to the need to shift attention between locations the present feature-based experimental design was generally more similar to the demands on neutrally cued trials in the spatial cueing task. In both cases, participants received no spatial information about the upcoming target. Still, the obtained fMRI data pattern more closely resembles that of spatially directed (valid cueing) rather than neutrally cued trials in our spatial cueing study^9^, possibly because of early attentional capture mechanisms interrupting a global search mode^75^. In line with similar behavioural data patterns across both studies, this suggests that the same regions were recruited to adjust the attentional goal in response to flanker interference on valid trials.

However, we need to account for the fact that there were no suprathreshold clusters in the spatial experiment in the invalid INC > CON contrast: We believe that the invalid condition plays a crucial role. Both study designs involved violations of expectations on invalidly cued trials (unexpected location; unexpected colour). Consequently, there were presumably no INC > CON clusters visible in SPL with invalid cueing in general: First, with spatial cueing, goal adjustments may have been implemented by shifting the attentional window, and second, during feature-based attention the attentional task set, i.e. the target colour was updated. We assume that both processes activated SPL and MFG, i.e. these structures were involved even on invalid CON trials.

Common activation of SPL and MFG on valid trials in the present studies (conjunction of INC > CON contrast over spatial and feature-based attention) and additional clusters with invalid feature-based attentional cueing in bilateral frontal cortices foster our interpretation of a domain-independent cortical network for attentional shifting processes. Likewise, these regions show common activation during shifts of attention both with spatial and feature-based attention in the literature (shifts between locations or between colours^36^. It is therefore likely that SPL and MFG not only respond to spatial orienting but also are more generally involved when the currently maintained attentional goal needs to be updated^36^. The fact that the discussed fronto-parietal structures demonstrated higher BOLD signals on INC compared with CON trials with valid cueing in both experiments points to enhanced efforts to overcome conflict on valid trials. There was possibly an early perceptual mismatch detection between flankers and targets. Thus, spatial focusing mechanisms potentially increased in both experiments, so that validly cued INC trials yielded higher activation levels than CON trials in fronto-parietal structures.

Apart from putative top-down activity for orienting overlapping between attention types, both studies also showed activity related to bottom-up processes. Thus, not only colour cueing (see previous paragraph) but also location cueing led to activity in portions of superior temporal gyrus (x/y/z: −40/−48/13) that are in line with TPJ as reported in the literature^71^ (; x/y/z of mean left TPJ Talairach peak coordinates: x/y/z: 57/−54/24; RMS distance: 21.1 mm). Embedding this pattern into existing models of attention networks (e.g.^13,14^), it is likely that the more salient incongruent stimuli initiated control signals through communication routes between TPJ and FEF. That is, TPJ may have been more active on valid INC trials in both experiments due to feedback signals to lingual gyri during detection of the perceptual mismatch on INC trials. Subsequent signals from TPJ to the dorsal network may in turn have initiated increased focusing as reflected in fMRI clusters in SPL and FEF. Paralleling these findings, when directly comparing our studies (conjunction analysis) we observed largely overlapping fronto-parietal networks between attention types (feature-based; spatial) for focused attention (valid cues) and reorienting (invalid cues) but also a hierarchical activation pattern^90^. In particular, spatially focused attention elicits higher BOLD signal increases in posterior regions (including left IPL/SMG, bilateral precuneus, and right middle temporal gyrus/middle occipital gyrus) than both feature-based attention and spatial reorienting. Therefore, dorsal and ventral fronto-parietal structures seem to be recruited for visual attention mechanisms, whereas more posterior brain structures specifically manage spatially directed attention. Hierarchical attention signals for spatial over feature-based attention have been reported elsewhere^91^. This fits with the observed relative voltage negativities of INC compared with CON trials within the N200 time window on valid trials and was initially hypothesised for the present study based on an analogous pattern in our spatial study.

Concerning the source analysis data, several of the RSs that were identified in the present study were also evident in the spatial cueing study^9^. For example, the RSs in left precentral gyrus demonstrated identical coordinates across studies. As reported above, a RS derived from peak coordinates in IFG and postcentral gyrus in the present study was near a RS in left IFG in that previous study. Given the low spatial resolution of SA^63^, these RSs are considered to be comparable. Moreover, sources in left ACC (both experiments) were in close proximity (RMS distance: 5.1 mm), as were sources in right postcentral gyrus in this study and right inferior parietal lobule in the spatial study (RMS distance: 20.9 mm). In addition, the sources seeded in bilateral lingual gyri and based on the colour localiser of the present study demonstrated coordinates adjacent to the RSs that were fitted based on principle component analysis in Siemann *et al*.^9^ (RMS distances: left lingual gyrus RS = 4.6 mm; right lingual gyrus RS: 7.6 mm). Finally, a present RS located in left parietal lobe showed similar coordinates to a RS in left middle temporal gyrus found in our previous study (RMS distance: 16.8 mm).

### Domain-specific mechanisms

Directly contrasting the fMRI flanker effects (INC > CON) between our two studies showed no domain-specific clusters when looking for activity specific to spatial cueing (spatial > feature-based). By contrast, the reverse comparison (feature-based > spatial) led to suprathreshold activity in left middle temporal gyrus, cuneus, and lingual gyrus. Both data sets were further compared in a conjunction analysis to extract overlapping activity patterns. The results show that the same structures that were more active in the present feature-based cueing study also seemed to be involved irrespective of cueing type. Therefore, while temporo-occipital areas executed interference processing in both tasks, the associated activation was further enhanced when feature-based cueing was used. This finding is in line with the aforementioned idea that attending to features required more resources than commonly reported in the literature (e.g.^14,24^) or in the same design with spatial attention^9^. Apparently, there is not only domain-specificity with respect to regional differences between attention types, but also concerning signal intensity. Likewise, Zani and colleagues^60^) found task-specific early attention effects using EEG (for a debate on C1 attention effects see ref.^92^). Furthermore, these effects suggest a more demanding processing during non-spatial compared to spatial selection^93^.

The source analysis reveals further domain-specific activity dynamics: RSs that were analogous in both experiments (ACC and precentral gyrus) demonstrated attention type specific temporal sequences of source waveforms. In our spatial cueing study, valid spatial cueing was first associated with INC > CON effects in precentral gyrus and subsequently in both RSs. This pattern may reflect the initial maintenance of two response alternatives (precentral gyrus) without conflict on validly cued trials due to early focusing mechanisms. Later INC > CON differences in both RSs (precentral gyrus and ACC) promote the idea that both response channels were reactivated after selection of the correct response when the attentional window broadened.

On the other hand, there were apparently stronger conflict signals originating in these RSs (ACC and precentral gyrus) when using spatial cues compared to feature-based cues during invalid trials. Hence, while early source waveform differences in both ACC and precentral gyrus were observable with spatial cueing potentially due to conflict detection, there were only late differences originating in the precentral gyrus RS in the feature-based experiment. This may be caused by control mechanisms originating in IFG, as there was enhanced source activity towards INC stimuli starting relatively early (~180 ms) in the present study. Due to an initial global search mode, the flanker stimuli potentially triggered control signals from IFG to ACC in preparation of the conflict. Later effects originating in precentral gyrus may therefore reflect conflict during response selection. In our spatial study in turn, increased responses arising in the IFG RS were also observable, but these occurred later than in the present feature-based cueing study, possibly during conflict resolution. Accordingly, ACC and IFG demonstrated simultaneous source waveform differences followed by enhanced activity originating in precentral gyrus when using spatial cueing, which hints at response conflict processing. The suggested multiple roles for IFG are consistent with a recent study that delineated inferior frontal cortex for signalling top-down attentional control from inferior frontal junction for reactive motor inhibition processes^94^.

### Critical reflections and limitations of the studies

There are some methodological issues that leave open alternative explanations of the results and limit the generalizability of the present findings.

First, due to differing source models, the present evaluation only provides an indirect comparison between studies. Even though the discussed sources (ACC; precentral gyrus; lingual gyri; IFG) were in close proximity to each other, each RS’s waveform pattern is contingent upon the contribution of all other sources^95^. Hence, additional sources that were not discussed here may have affected the observed source waveform patterns depending on the respective source model.

Second, in the comparison of the present flanker studies with spatial and feature-based attention, distinct modes of conflict processing are postulated. However, the bootstrap 95% confidence interval in the RS in ACC on invalidly cued trials was much larger when cueing colours compared to locations, pointing to larger variabilities between individual source waveform patterns. This was particularly evident between 500–600 ms in the colour cueing experiment when the RS in precentral gyrus demonstrated congruency effects. Therefore, conclusions based on an absent difference between conditions in ACC in that study cannot be drawn unequivocally. Source waveforms originating in the precentral gyrus RS demonstrated INC > CON differences around 580 ms, hinting at a response conflict comparable to that with valid cueing. It is therefore conceivable that ACC demonstrated simultaneous source waveform differences as well in the feature-based cueing study, but only at a subthreshold level due to high inter-individual differences. ACC appears to be active during strategic adaptations based on previous trial history^44^ and is additionally involved in within-trial adjustments^96^. Such on-line modulations are apparently contingent on individual response styles, leading to increased variability of signals arising in ACC^97^.

Third, a past study on domain-specificity delivering results compatible with our studies relied on multi-voxel pattern analysis to extract differences between attention types^36^. On a whole-brain level, overlapping and apparent domain-general structures for feature-based and spatial attention were obtained. More sophisticated data analysis thus informed about mechanisms in the data patterns not visible using standard methods alone. As a substantial difference between Greenberg *et al*.^36^ and our studies, our main interest was the effect that both attention types (feature-based; spatial) have on interference processing. Our results therefore focussed on the flanker contrast (INC > CON) instead of attention effects (location; colour). For that reason, we believe that the similarity between the overlapping brain regions across our studies and those reported in Greenberg *et al*.^36^ support the idea that domain-general attention mechanisms assisted during conflict processing. In turn, we do admit that our macro-analytic data analysis approach leaves open alternative explanations.

## Conclusions

Comparing the effects of different attention modes (feature-based; spatial) on flanker interference processing yielded a similar recruitment of fronto-parietal brain structures but distinct conflict processing mechanisms.

Overlapping activity between experiments in the INC > CON contrast with valid cueing in SPL and putative FEF points to a general dorsal fronto-parietal network for top-down control of conflict processing. While correct spatial attention probably involved early attentional window adjustments to suppress flankers, globally directed feature-based attention may initially have involved attentional captures to the stimulus location followed by similar focusing processes as with spatial cues. Flexible attentional window adjustments in both experiments are additionally corroborated by overlapping activity in TPJ, which possibly triggered violations of expectations to FEF with respect to both locations and colours. Invalid cueing yielded no congruency effects in SPL or FEF, probably because activity in these regions was generally enhanced due to updating of the attended colour (feature-based cueing) or attentional reorienting (spatial cueing). This may in turn have shadowed additional brain activity towards INC flanker stimuli, which parallels a postulated ceiling effect on invalid trials in the behavioural data leading to similar flanker effect sizes as on valid trials. Similar to overlapping neuronal activity patterns in fronto-parietal regions across studies, the same visual brain regions were apparently recruited reflecting activity due to feedback projections stemming from the fronto-parietal network.

Conflict processing additionally activated ACC and precentral gyrus with valid feature-based cueing, and source waveform data from these regions hint at the response stage of conflict resolution. The reason for this finding might be a more difficult response selection because of enhanced initial activation of competing response channels in comparison with the spatial cueing study because of an initial global search mode associated with feature-based cueing.

## Electronic supplementary material

Supplementary Figures S1 and S2
