# Supplementary material for: The effect of feature-based attention on flanker interference processing: An fMRI-constrained source analysis
Source: Sci Rep. 2018 Jan 25;8:1580. doi: 10.1038/s41598-018-20049-1 (PMC5785471; doi:10.1038/s41598-018-20049-1)
Supplement: Supplementary file 1 — Supplementary Figures S1 and S2 [file 41598_2018_20049_MOESM1_ESM.pdf]

The effect of feature-based attention on flanker interference processing: An fMRI-constrained source analysis

Julia Siemann, Manfred Herrmann and Daniela Galashan

Supplementary Figures S1 and S2

Supplementary Figure S1:

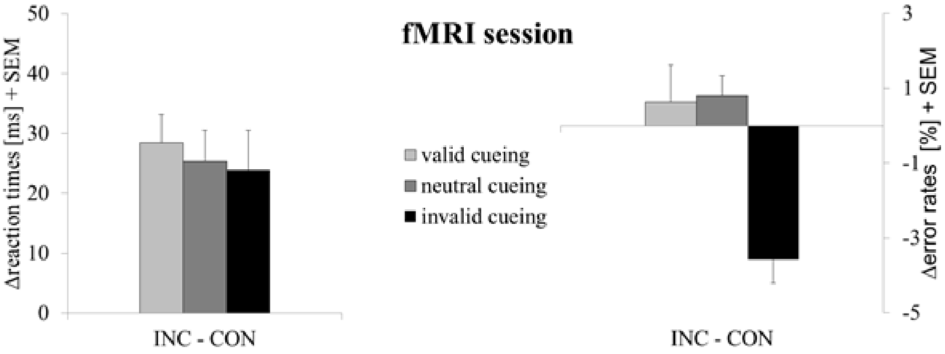

Behavioural data of the fMRI session: Differences (Δ) of the reaction times (left) and percent error rates (right) between incongruent (INC) and congruent (CON) flanker conditions during valid (light grey), neutral (medium grey), and invalid cueing (black). Error bars represent standard error of the mean (SEM). N = 21

Supplementary Figure S2:

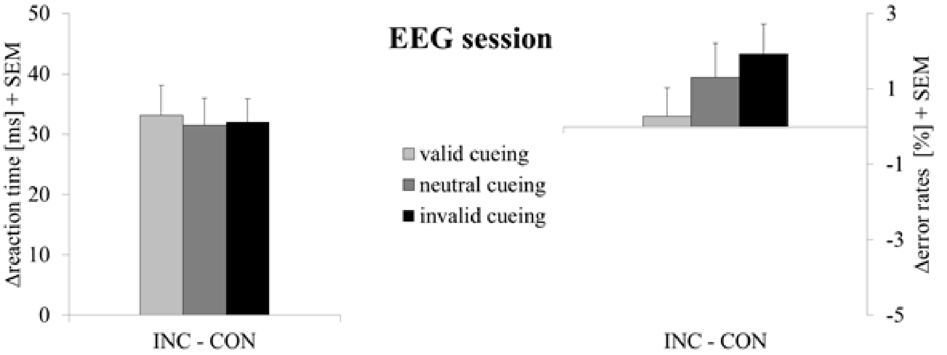

Behavioural data of the EEG session: Differences (Δ) of the reaction times (left) and percent error rates (right) between incongruent (INC) and congruent (CON) flanker conditions during valid (light grey), neutral (medium grey), and invalid cueing (black). Error bars represent standard error of the mean (SEM). N = 21.
